# The Application of ‘Elite Interviewing’ Methodology in Transdisciplinary Research: a Record of Process and Lessons Learned during a 3-Year Pilot in Urban Planetary Health Research

**DOI:** 10.1007/s11524-021-00542-1

**Published:** 2021-05-14

**Authors:** Gabriel Scally, Daniel Black, Paul Pilkington, Ben Williams, Janet Ige-Elegbede, Emily Prestwood

**Affiliations:** 1 University of Bristol, Bristol, UK; 2 Daniel Black + Associates | db+a, Bristol, UK; 3 University of the West of England, Bristol, UK; 4 University of Birmingham, Birmingham, UK

**Keywords:** Elite interviewing, Methodology, Urban development, Planetary health, Upstream

## Abstract

This paper sets out the rationale and process for the interviewing methodology utilized during a 3-year research pilot, ‘Moving Health Upstream in Urban Development’ (UPSTREAM). The project had two primary aims: firstly, to attempt to value economically the health cost benefits associated with the quality of urban environments and secondly, to engage with those in control of urban development in the UK in order to determine what are the barriers to and opportunities for creating healthy urban environments, including those identified through the utilisation of economic valuation. Engagement at senior level with those who have most control over key facets of planning and development implementation—such as land disposal, investment, development delivery and planning permission—was central to the approach, which encompassed the adoption of ‘elite interviewing’, a method developed in the USA in the 1950s and used in the political sciences but relatively unutilized in the health and environmental sciences [1]. Two rounds of semi-structured interviews were undertaken with 15 senior decision-makers from the UK’s main urban development delivery agencies, both public and private. The ‘elite interviewing’ approach successfully enabled the UPSTREAM project to capture and analyse the information received from the interviewees, all of whom held influential or leadership posts in organisations that are important actors in the process of planning, developing and constructing the built environment in the UK. Having academic and practitioner research leads on an equal footing created some minor tensions, but it also appeared to strengthen the rigor of the approach through a broad knowledge of context ‘in-house’. This form of co-production at times challenged academic traditions in qualitative analysis, but it also appeared to build trust with interviewees and provided greater clarity of the real-world context under investigation. Findings from this study are written up in a separate paper.

## Introduction

The pilot ‘Moving Health Upstream in Urban Development’ (UPSTREAM) was funded by the Wellcome Trust under their Our Planet Our Health (OPOH) programme, which supports researchers to take on the challenges that (a) food systems, (b) increasing urbanisation and (c) climate change pose to our health. OPOH aims to provide strong evidence for action so that policymakers, businesses and the public can make more informed decisions on things that affect the environment and health [2]. It was led by a steering group of academic and external practitioner-researchers and had two primary aims:

1. To develop the use of economic valuation in understanding the quality of the urban environment and its measurable impact on human and planetary health [3]
2. To understand from those in control of the urban development in the UK what the main barriers and opportunities are in creating healthy urban environments

This paper sets out the rationale for the pilot, providing a brief overview of the evidence linking urban environments with human and planetary health outcomes, alongside a description of the challenges in enabling substantive change in this area (within the UK specifically, but with lessons of relevance to geo-political and urban development contexts with shared systems; broadly, European and other ‘Western’ OECD countries). We describe the underpinning considerations vital for effective interviewing in this context, the sample of interviewees, why and how they were selected and the interviewing and analysis processes undertaken. The strengths and limitations of the approach are explored, along with description of how we sought to overcome impediments and how we propose to develop further this approach.

The interview findings, key discussion points and priority research areas are presented in a separate paper [4].

## Background and Project Rationale

Urban environments and public health have a long, shared history. Although the specifics of population health risk have changed significantly since the nineteenth century sanitary revolution, the rise of non-communicable diseases (e.g. cancers, diabetes, respiratory illnesses) and global environmental risk factors (e.g. flooding, heat waves, resource depletion, migration) are due in no small part to poor urban environments and linked behaviors, and they are placing increasing stress on our human and planetary life support systems in the UK as elsewhere across the urbanising world [5–10].

Significant research has been undertaken on healthy and sustainable urban planning and design over recent decades and is represented widely within the ‘grey literature’ [8, 11–20].

**Fig. 1:**
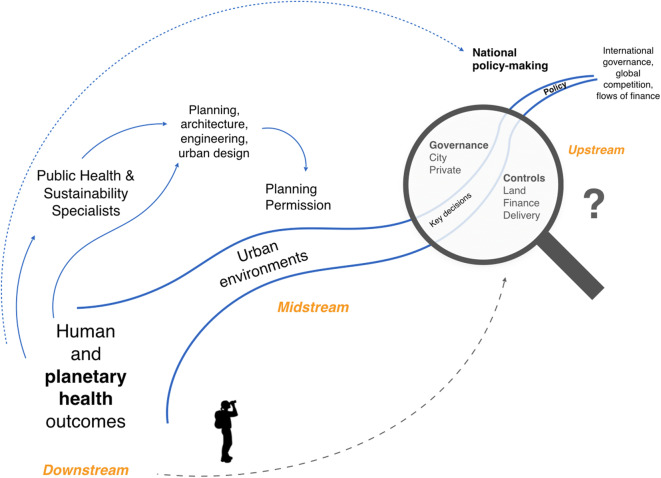
An illustration showing (a) the different activities in urban development and which decisions and actors take precedence, (b) the relative familiarity of the health and urban research community with downstream and mid-stream activity and (c) the relative disconnect between those aware of public and planetary health outcomes downstream and those responsible for critical decision-making far upstream.

We see planning and design of the built environment as being in the ‘midstream’ of the urban development process, as opposed to those root cause decisions made further upstream at the city level and above, which include key points of influence such as ‘disposal’ (sale) of land, investment controls and control of delivery process [21, 22]. Figure 1 illustrates the range of different actors, disciplines and decision areas along the urban development stream of activities.

**Box 1:** Key points relating to elite interviewing

• The entire approach should be conducted in a highly professional manner • Interviews should invariably be conducted by the most senior investigators • A limited number of key probes for areas in which information is sought should be at the core of the interview • Effective note-keeping prior to, during the course of, and after the interview is vital, as non-textual learning may be very important • Rapid analysis and coding of the output of the interview is important so as to inform and develop the process and the areas of enquiry as the study proceeds • Time should be set aside to prepare and rehearse for the interviews • Preparation should include a background brief on each interviewee and their organisation	

Despite the UK now having globally renowned expertise and workforces in the built environment professions, our towns and cities continue to be polluted and dominated by cars, and buildings and public spaces are often of poor quality are lifeless [8, 23–26]. Behavior in urban environments is overwhelmingly directed towards consumption and unhealthy eating and drinking. This, plus limited opportunities for accessing nature which means we are increasingly disconnected from the natural world, is impacting significantly on our physical and mental health [8, 13, 27].

There are increasing calls from the public health practice and linked academic communities to examine factors upstream, not just at the stage of design of the built environment itself [28] but to consider the ‘commercial determinants of health’ and issues such as global flows of human resources and capital [29–31].

We have already described the further challenge areas, which we suggest include valuation failure and specifically market and government inability to internalize current and future costs to human and planetary health; the disconnection between research and practice resulting in misunderstanding, an underutilized knowledge base and limited impact; and the sheer complexity of actors and processes involved along with other factors in influencing urban development decision-making [32]. We proposed specifically the need for engagement and co-production with those in control of the development delivery processes, alongside the need for innovative new process for balancing engagement with community representatives affected by these upstream decisions.

Building on this project rationale, central to our approach were the following key aspects: first, the use of emerging non-market economic valuation methods to support decision-making more informed about ‘external’ costs; second, the central role of experienced practitioner-researchers as a core part of the research team leadership, responsible for bridging the gaps between the worlds of academia and practice in this specific area; third, an unbounded approach to investigation based on consideration of whole systems (and other linked systems); and finally, a focus within the pilot on senior decision-makers and on methodological approaches that enable effective data collection and analysis given the inherent constraints and factors influencing engagement with those with limited time [21].

Implicit within this starting position too are the notions of inter- and trans-disciplinary working, or at the very least the need for co-production with a wide range of stakeholders. This is an area very familiar to those who work in urban planning and related fields (e.g. ‘masterplanning’, urban design review, health and sustainability assessment) where public participation and community consultation, engagement and involvement have long been practiced and are widely required (despite it now being widely recognized as paying ‘lip service’ to the notion of genuine community involvement) [33–38]. There is now considerable literature on the benefits of co-production in research and across many fields—e.g. healthcare (‘patient and public involvement or engagement’), law or product design—and using a wide variety of processes and with a range of communities, most notably perhaps the lay public, though also with targeted sector-specific or topic-related groups (e.g. civil service, local government, consumers, commercial partners) [39–44]. There is also a ‘dark side’ to co-production in that it can often be perceived as a universal good to aspire to greater levels of inclusivity, which in and of itself presents its own challenges (e.g. raising of expectations, poor understanding of who to involve, ‘consultation fatigue’, disconnect between involvement plan and resource available, not keeping your ‘eye on the prize’ of reduction in NCD) [45–47]. Finally, how we approach co-production has implications beyond research design to broader issues of research governance and the structural challenges across the research ecosystem. Key points for consideration appear to include, e.g. overspecialisation within academia and the prevailing disconnection between academia and the real world and a growing demand for ‘knowledge brokers’ and ‘blended professionals’, alongside a similar challenge to co-production: the ‘dark side of knowledge brokerage’. [21, 48–53]

## Objectives and Phases

The main objectives were as follows:

1. To demonstrate to decision-makers the hidden costs of poor-quality urban development
2. To test what impact monetary valuation of health outcomes may have on decision-makers
3. To identify the barriers facing (and opportunities open to) decision-makers
4. To validate this taxonomy of barriers and opportunities and have endorsed the resulting strategy
5. To disseminate the results nationally and internationally

**Fig. 2:**
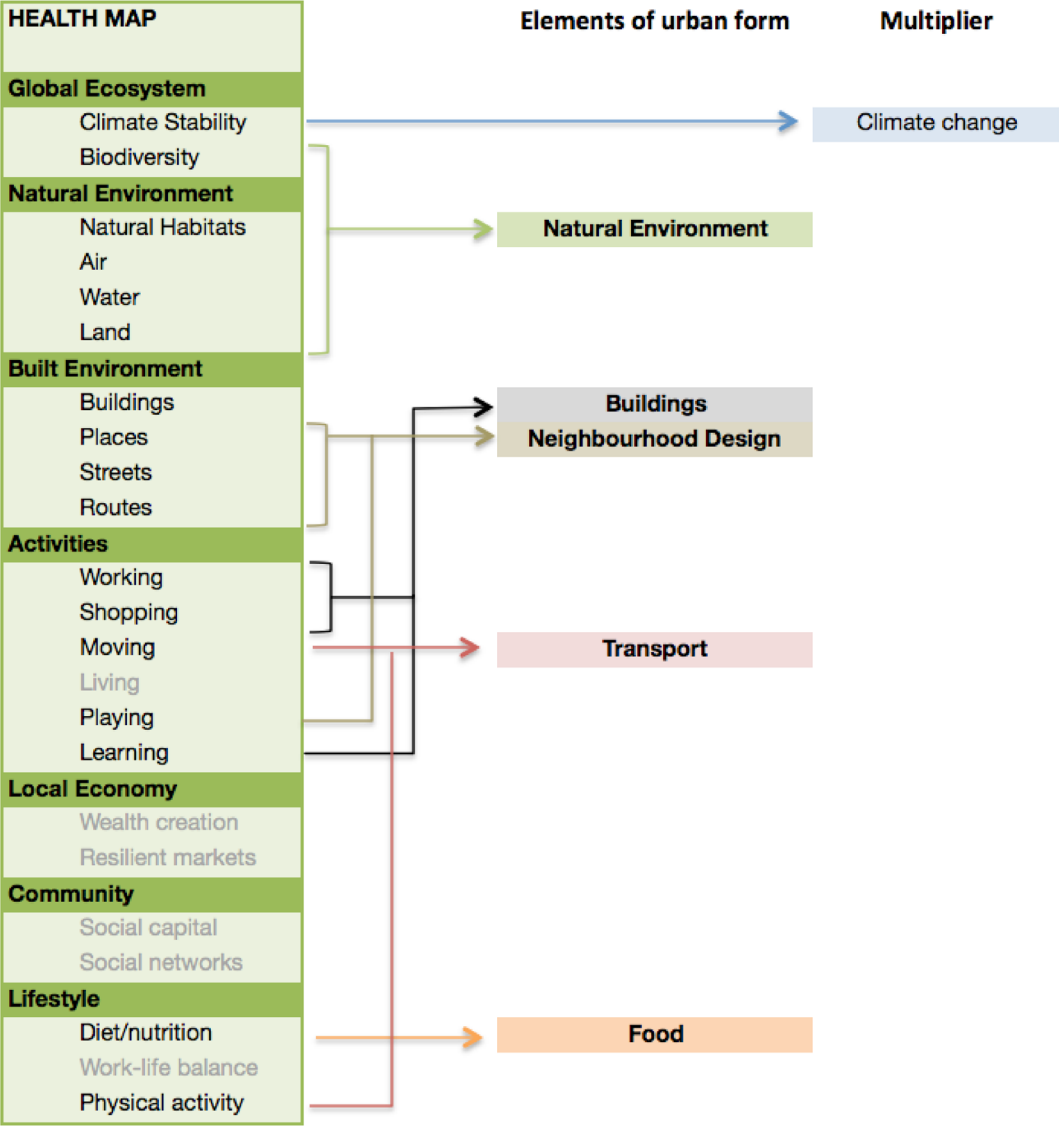
The five areas of search used in the umbrella review were derived from the Health Map, a graphical prompt that lists primary determinants of health linked to the built environment. It was used as a stem checklist of categories and was compared against four other health and built environment tools. Climate is revealed as a ‘multiplier’ factor across all five

In order to achieve these objectives, the project was split into three balanced and overlapping phases:

1. An umbrella review (a systematic review of review-level evidence) including over 200 studies from the health literature examining associations between the urban environment and health outcomes splits into five main search areas: buildings, transport, natural environment, neighborhood design and food (Fig. 2). Evidence in each area was obtained from a systematic search of relevant electronic databases and other online sources, using specified keywords, with quality assessment of identified studies and narrative synthesis of findings. The areas of search were derived through a comparative exercise mapping categories from five different assessment tools: (i) the Health Map, a graphical prompt listing the primary determinants of health linked to the built environment, was used as a stem checklist; (ii) the Vancouver Health Impact Assessment Toolkit; [54, 55] (iii) BREEAM Communities; [56] (iv) HUDU Rapid HIA [57]; and (v) the Egan Review. [58] Climate change was seen as a ‘multiplier’ that was factored across all other categories.
2. A valuation of the urban-health data and associated economic cost-benefits. [3]
3. Two rounds of semi-structured interviews with 15 senior public and private sector decision-makers from the urban development world. [4]

## Elite Interview Method and Sampling

The study used a qualitative ‘elite interviewing’ approach consisting of two rounds of in-depth semi-structured interviews (both face-to-face and over the telephone) with 15 interviewees. For this research, the use of the term ‘elite’ was consistent with its commonly used definition as a member of a group of persons exercising a major share of authority or influence within a larger group or organisation. Elite interviewing has almost exclusively been applied at top levels within coherent occupational or professional groupings, e.g. US politicians and healthcare executives, yet its flexibility and underpinning theory fit well with the multi-sectoral group of interests represented amongst our target interviewees. [59–61] Our proposition was that with the right transdisciplinary team and approach, it can overcome, in a demanding multi-sectoral arena, both methodological issues such as power relations as well as practical issues experienced by junior researchers such as gaining access, establishing trust and dealing with interpersonal challenges. [62, 63]

While it can be categorized as a type of semi-structured interviewing methodology, elite interviewing demands a nuanced approach to preparation, implementation and data analysis, which is crucial for effective research (Box 1).

The elite interview requests were targeted at individual senior executives from the public and private sector, who were likely to have a full understanding not only of their own organisations but how their organisations fit within the wider system (Table 1). Candidates for interview were identified mainly through existing practitioner networks identified in purposeful discussion amongst the multidisciplinary research team members. As UPSTREAM had been actively communicating its research activity, one interviewee came via their expression of interest on LinkedIn. All interviewees expressed a pre-existing interest in the research area (there had already been considerable work industry-wide on sustainability issues, and urban health was a growing area of interest). They did not receive a stipend.

**Table 1:** Overview of interviewee sample showing sectors, organisations, interviewee numbers and positions within organisations

Sector	Organisation	No. of interviewees	Position within company
Private	• Volume House-Builder • Developer/Asset Manager • Regeneration JV • Investor Social Enterprise	6	Senior executives
		2	Sustainability/health specialists
Public	• City Council • District Council • Development Corporation	5	Senior executives
		2	Sustainability/health specialists

In choosing to interview those in the ‘elite’ category, a differentiation is drawn between those who have ‘interpretive power’ based on their knowledge and skill (the experts) and those who have ‘formative power’ because of their position in organisations and their direct involvement in, or proximity to, decision-making (the elite). [64] They were either at director or chief executive level in their organisation or in a position where they regularly controlled or influenced decision-making on urban development at the highest level. Some of the executives were supported in the interviews by the health/sustainability leads from their organisation. Of those interviewed, eleven were male and four female, the latter all in the public sector.

Defining those to be interviewed was a key task, and, as Littig has pointed out, sampling in elite interviewing ‘does not adhere to quantitative conceptions of representativity’. [64] The group was a purposive sample, hitherto unknown to the lead interviewer, and derived mainly through existing networks, although one interviewee expressed their interest in taking part following a notice posted on LinkedIn. The justification for this was the relative inaccessibility of senior decision-makers in this field, which takes in to account the ‘distance’ between researchers and practitioners on the one hand and the public health sector and our target sample group (developers, investors and landowners) on the other.

It is worth underlining that the focus was on the planning and management of urban areas, including large-scale mixed-use urban development projects, i.e. buildings primarily—residential, employment, retail, leisure—and supporting infrastructure (streets, public transport, green/blue infrastructure, community amenities), as opposed to specific large-scale infrastructure such as new rail, road, energy or telecommunications projects.

**Fig. 3:**
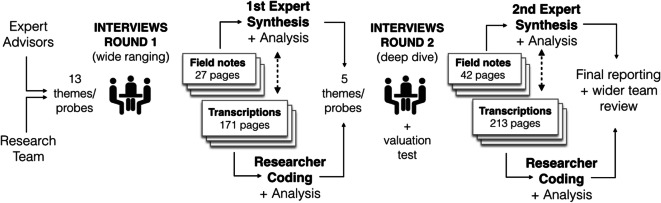
Flow chart illustrating process of iterative interview theme co-development and analysis. The first round of interviews started with thirteen themes and associated probes. Five themes were selected for a ‘deeper dive’ in round two. In the final analysis, these themes were combined into eight main themes in the industry report and interview findings paper

The first round of interviews took place between June 2017 and November 2017, and the second round took place between June 2018 and September 2018. The first round of interviews was undertaken using a framework of 13 thematic areas and associated probes (Fig. 3). The research team developed the first-round thematic areas and probes following informal interviews with four senior independent advisors with long experience in key areas of urban development practice: real estate, city government, estate agency and volume house building. Five second-round thematic areas and probes were identified by the research team through internal research group analysis and reflection post hoc of the field notes and coded transcripts. These were intended to allow key areas identified in the first and subsequent analysis to be explored in greater detail. Approximately a third of the time in the second-round interviews were allocated to discussion of the economic valuation findings and two-thirds to deeper exploration of key themes identified.

**Fig. 4:**
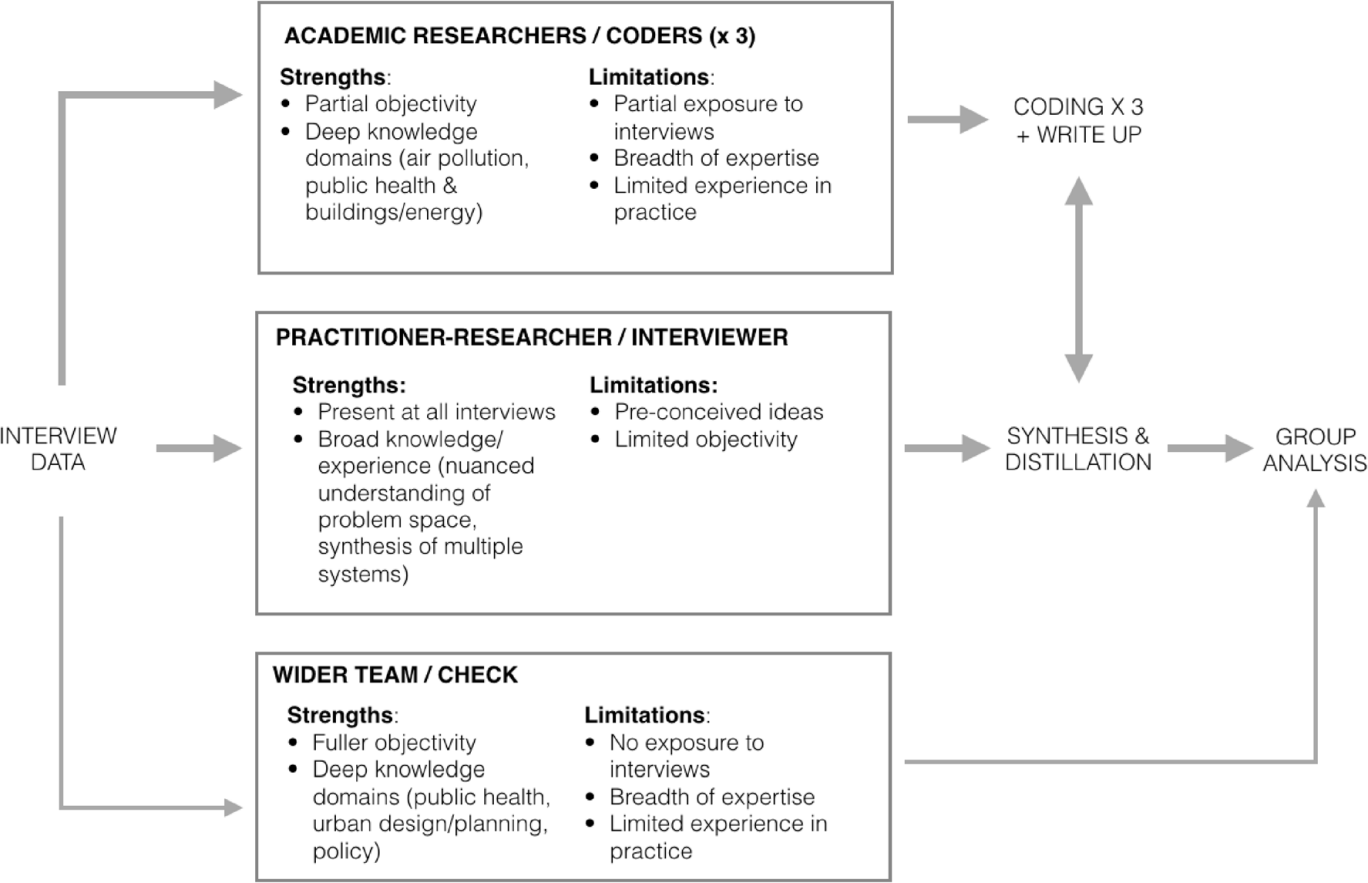
Flow of ‘three-pronged’ analysis process aimed at shoring up shortcomings in each individual approach, setting out strengths and limitations of each

In the analysis, we combined two main approaches: (1) synthesis by the lead interviewer who was present at every interview drawing from the interviews, field notes and corroborating against the transcriptions and (2) coding (using NVIVO) of transcriptions by three researchers who had been individually present at one or more interviews, using Braun and Clarke’s framework for thematic analysis. [65] A third point of validation was sought from the wider internal project team when presenting back the summary of the synthesized analysis (Fig. 4). Informants were not part of this final reporting stage but did receive transcriptions for comment and correction during the process of theme development and analysis. The study received ethical approval from the University of the West of England. At all stages of data collection and analysis, data was stored securely in accordance with the University of the West of England’s data management procedures. Quotes were anonymized in outputs, so as to preserve anonymity for participants.

## Discussion

The decision to adopt the elite interviewing approach resulted from an early realisation that we would need an approach to semi-structured interviewing that could take in to account the contextual factors set out above (e.g. wide range of subject matter, the need for broad practitioner expertise to facilitate discussion, time limitations). It was not only judged to be well suited to our purpose but would also be critical in ensuring we could satisfy the aims and objectives of the pilot.

Our experience during the study and the richness of the data collected appears to have born these judgements. To give one example where the approach appeared to bear fruit, all interviewees seemed, and their behavior supported this view, to be stimulated and engaged by the conversations, which led to longer time in conversation, considerably more data and greater levels of ongoing engagement. Each interview was scheduled to run for an hour, and though one was shortened due to limited time availability of the interviewee, most ran substantially over the time agreed despite interviewees saying initially they needed to keep to time. We believe this would have been far less likely if the interview had been more tightly structured, less conversational and more question focussed (i.e. less intellectually stimulating and, potentially, perceived as less relevant).

Another example of the benefit of this nuanced approach was the nature of the interpretation and analysis. There was a difference of opinion between the academic researchers and the practitioner-researchers as to the best approach. The academic researchers understandably sought to employ a realist approach whereby the coding has primacy, [66] and should direct the analysis, given that it is drawing directly from the raw data using a widely accepted method and is corroborated by three different researchers. In contrast, a single individual referring to field notes to draw out findings, even if they were then corroborated against the verbatim transcriptions, is understandably accorded far less weight. From the practitioner-researcher perspective, however, as encompassed in the elite interviewing methodology, there is a ‘significant value in the non-textual learning’, and there is a need for substantial experience in order to effectively undertake the research. As Black (2006) argues, ‘how can words fully express the meaning inherent in our observations, personal interviews and pictures when so much of it is subtle, hidden and contextually bound?’ [67]

In order to support both positions—i.e. ensuring appropriate checks are in place while also acknowledging the primacy in this occasion of practitioner insight—the research team decided to further triangulate the approach by adopting a ‘three-pronged’ analysis led by the practitioner-researcher but checked firstly against the three academic researcher coding and, secondly, then checked that synthesized analysis with the wider team. Figure 4 sets out the various strengths and limitations of each approach and how, together, they were made to be mutually supportive.

We believe that the co-production between the academics in the project and the practitioner is a key learning output from the research. While it challenges traditional academic approaches to qualitative analysis, we feel that in the context of elite interviewing, it strengthens analysis by combining academic rigor with practitioner expertise, leading to outputs that reflect more fully the real-world context of the area under study.

There has been considerable critique of the elite interviewing approach. [61, 68, 69] In addition to the recognized limitations inherent in qualitative interviewing approaches (e.g. small sample size and lack of ‘triangulation’; expertise, subjective bias and influence of researchers; reporting framework and limitations), interviewing ‘elites’ presents a number of additional potential constraints (e.g. time availability of interviewees, knowledge of lead interviewer and associated bias, [66, 70–73] knowledge of research coders and interview bias, getting balance right between). [59, 61, 74] These critiques have proven valuable to us in helping avoid some of the potential issues, and our operational methods have sought to avoid or mitigate many of these issues arising.

An issue to address when conducting elite interviewing is the factor that might influence the willingness of elite individuals to participate. We did not use any form of financial incentive to encourage engagement in our research. Instead, we found that participants were motivated to engage through a pre-existing interest in the sustainability and health agendas. We also feel that an important factor in our success in recruiting ‘elite’ participants was to have a lead interviewer who was an experienced practitioner in the field, who had a broad understanding of the sectors involved, had established expertise in at least one cognate discipline and could ‘speak their language’.

In common with other qualitative approaches, the question of external validity needs to be considered. Although it is not possible to prove the external validity of our findings, we believe that the participants did represent a spread of actors across the field under study. And while it is possible that knowledge, experience and attitudes towards health and development amongst those who agreed to participate in our research may differ from others in the field (particularly given our comment in the previous paragraph), we did not observe any patterns that might point towards possible participant bias (such as high levels of refusals to participate in the research amongst those we approached).

During the carrying out of the research, we have also had researchers, both within and outside our wider team, questioning the use of the word ‘elite’ (both as an accurate descriptor in itself and due to it being seen as ‘elitist’, focusing attention on the privileged few), as well as dismissing it as either too constrained or little different from standard interviewing methods. It is clear that the term ‘elite’ is uncomfortable for many people and carries with it strong connotations of class, gender and racial inequality. However, the methodology of ‘elite interviewing’ as a means of exploring key research questions with people who have power has proven valuable in research, notably in political science, and we have found it to be appropriate and effective in the multidisciplinary context. As for the specific title ‘elite interviewing’, we would concur with the sentiment as expressed by Davis Riesman in 1956:

I am not happy with the term “elite,” with its connotations of superiority. Yet I have found no other term that is shorthand for the point I want to make, namely that people in important or exposed positions may require VIP interviewing treatment on the topics which relate to their importance and exposure. [75]

## Conclusion

We have successfully developed and employed the ‘elite interviewing’ methodology, which originated in the world of political science in the USA, to a multi-disciplinary group of decision-makers with considerable influence over the nature of urban development in the UK and thus it’s capacity to contribute to the solution of problems associated with the causation of major non-communicable diseases burdens in society. We maintain that this approach has been crucial to the successful carrying out of our research, giving us all-important flexibility in suiting the research implementation and analysis to task. While we acknowledge its limitations, which need to be made clear when presenting method and findings, we suggest the benefits can, if the technique is applied appropriately, far outweigh the shortfalls. We will be employing and developing the methodology further in our new 5-year research programme funded under the UK Prevention Research Partnership: ‘Tackling Root Causes Upstream of Unhealthy Urban Development’. [76]
